# Melatonin as an Antioxidant Agent in Stroke: An Updated Review

**DOI:** 10.14336/AD.2022.0405

**Published:** 2022-12-01

**Authors:** Junjie Wang, Shiqi Gao, Cameron Lenahan, Yichen Gu, Xiaoyu Wang, Yuanjian Fang, Weilin Xu, Haijian Wu, Yuanbo Pan, Anwen Shao, Jianmin Zhang

**Affiliations:** ^1^Department of Neurosurgery, The Fourth Affiliated Hospital, International Institutes of Medicine, Zhejiang University School of Medicine, Yiwu, Zhejiang, China.; ^2^Department of Neurosurgery, The Second Affiliated Hospital, Zhejiang University School of Medicine, Hangzhou, Zhejiang, China.; ^3^Department of Biomedical Science, Burrell College of Osteopathic Medicine, Las Cruces, NM, USA.; ^4^Brain Research Institute, Zhejiang University, Hangzhou, Zhejiang, China

**Keywords:** melatonin, stroke, neuroprotection, oxidative stress, mechanism

## Abstract

Stroke is a devastating disease associated with high mortality and disability worldwide, and is generally classified as ischemic or hemorrhagic, which share certain similar pathophysiological processes. Oxidative stress is a critical factor involved in stroke-induced injury, which not only directly damages brain tissue, but also enhances a series of pathological signaling cascades, contributing to inflammation, brain edema, and neuronal death. To alleviate these serious secondary brain injuries, neuroprotective agents targeting oxidative stress inhibition may serve as a promising treatment strategy. Melatonin is a hormone secreted by the pineal gland, and has various properties, such as antioxidation, anti-inflammation, circadian rhythm modulation, and promotion of tissue regeneration. Numerous animal experiments studying stroke have confirmed that melatonin exerts considerable neuroprotective effects, partially via anti-oxidative stress. In this review, we introduce the possible role of melatonin as an antioxidant in the treatment of stroke based on the latest published studies of animal experiments and clinical research.

## 1. Introduction

A cerebrovascular accident, otherwise known as stroke, is a devastating disease that remains the second-leading cause of death, and the third-leading cause of death and disability combined globally [1, 2]. Stroke is generally classified as ischemic or hemorrhagic, and hemorrhagic stroke consists of intracerebral hemorrhage (ICH) and subarachnoid hemorrhage (SAH). After stroke onset, complicated pathophysiological processes are involved in the brain, such as excitotoxicity, oxidative stress, and inflammation, which may exacerbate brain injury and worsen the prognosis. Although these cellular events are potential therapeutic targets for stroke treatment, viable treatment options are unfortunately still limited [3].

Oxidative stress plays a pivotal role in the pathogenesis of both ischemic and hemorrhagic stroke. Originating from excess reactive oxidant accumulation, oxidative stress not only directly damages brain tissue, but also enhances a series of pathological signaling cascades, contributing to inflammation, brain edema, and neuronal death [4-6]. Therefore, optimal neuroprotective agents for treating stroke should target multiple pathological pathways, including oxidative stress, to counteract a series of secondary brain injuries. As an endogenous antioxidant with pleiotropic effects, melatonin has demonstrated neuroprotection in animal experiments by alleviating oxidative stress, inflammatory responses, and apoptosis [7, 8]. However, the confirmed clinical application of melatonin has primarily been applied to insomnia-related disorders [9]. As research of this molecule progresses, recent preclinical experiments and clinical studies have reported the role and effect of melatonin in stroke, which guides the exploration of underlying mechanisms, and may expand its clinical application.

Herein, we review the roles of oxidants/antioxidants and the damage of oxidative stress during both ischemic and hemorrhagic stroke. Furthermore, we summarize the physiological features of melatonin, especially the antioxidative property and the action pathways. We finally survey the current progress in preclinical experiments and clinical research to discuss the neuroprotective effects and practical applications of melatonin for stroke treatment.

## 2. Oxidative Stress and Stroke

### 2.1 Free Radicals and Antioxidative Systems

#### 2.1.1 Free Radicals and ROS/RNS

Free radicals are molecules or molecular fragments containing one or more unpaired electrons in atomic or molecular orbitals [10]. These highly reactive radicals can generally be divided into two main groups: reactive oxygen species (ROS) and reactive nitrogen species (RNS) [2]. Notably, some non-radicals (no unpaired electron), such as hydrogen peroxide (H_2_O_2_), organic hydroperoxides (ROOH), and peroxynitrite (ONOO^-^), are also classified as ROS/RNS [12, 13].

ROS/RNS is an umbrella term that encompasses numerous molecules with diverse properties. The most critical reactive species amongst the two big families are superoxide anion radical ( $O_{2}^{\cdot -}$), hydrogen peroxide (H_2_O_2_), hydroxyl radical (·OH), nitric oxide (NO), and peroxynitrite (ONOO^-^). Various sources can give rise to ROS, involving non-enzymatic sources and enzymatic sources. The non-enzymatic sources include Fenton/Haber-Weiss reactions (requiring free iron), as well as hemoglobin or myoglobin (releasing into extracellular fluid after injury) [14, 15]. The major enzymatic sources of ROS contain the electron transport chain (ETC) complexes in mitochondria, as well as a variety of oxidases, such as xanthine oxidase (XO) and NADPH oxidase (NOX), that are widely present in the endoplasmic reticulum (ER), peroxisomes, and cytosol [12].

$O_{2}^{\cdot -}$is produced mainly in mitochondria, is considered the “primary” ROS, and can interact with other molecules to further generate a series of “secondary” ROS [16]. $O_{2}^{\cdot -}$serves as a significant source of H_2_O_2_ through catalyzation by superoxide dismutase (SOD). Besides, $O_{2}^{\cdot -}$ can react with NO to form ONOO^-^, a potentially harmful species associated with inflammation [17]. H_2_O_2_ is a relatively stable ROS with multiple biological functions. In the presence of free iron, especially when an excess of superoxide releases free iron from iron-containing molecules, ·OH can be formed from H_2_O_2_ via Fenton/Haber-Weiss reactions. Moreover, ·OH is the most reactive and aggressive ROS, but has a relatively short half-life, and ·OH is perceived as a dominating player in oxidative damage, mainly due to the massive destruction of DNA bases and lipid peroxidation it causes[9, 10, 14]. In addition, nitric oxide (NO) can be generated via three isoforms of nitric oxide synthase (NOS): neuronal NOS (nNOS), endothelial NOS (eNOS), and inducible NOS (iNOS) [18]. NO usually exerts neuroprotective function due to its antioxidative and anti-inflammatory properties [11]. Interestingly, the beneficial effects of NOS can be reversed under the impact of cardiovascular risk factors. Under this condition, NOS can be converted into a dysfunctional $O_{2}^{\cdot -}$-generating enzyme, a process referred to as NOS uncoupling [15, 20].

As is well-known, ROS/RNS have dual roles as both deleterious and beneficial species [10, 21]. Oxidative eustress, considered the opposite of oxidative stress, refers to the positive effect of ROS/RNS associated with the normal physiological process [13]. The occurrence of oxidative eustress requires a physiological environment where these oxidants are maintained at a low/moderate level. For example, H_2_O_2_, at the desirable concentration (approximately 1-10 nM), can reversibly oxidize specific protein targets to mediate beneficial biological signaling effects, such as cell proliferation, differentiation, migration, and angiogenesis [12]. The physiological levels of H_2_O_2_ are important to preserve neuronal growth/guidance and stem cell function. Insufficient H_2_O_2_ levels lead to impaired development and regeneration of neurons, as well as a decreased propensity of stem cell proliferation and differentiation. However, a pathological increase in H_2_O_2_ levels induces axodendritic degeneration and collapse, stem cell senescence, exhaustion, or even death [12]. Therefore, the supraphysiological concentration of ROS/RNS can lead to the nonspecific oxidation of proteins and other biomolecules, resulting in a pathological process known as oxidative stress [12, 23]. For example, stroke-induced progressive insult of tissues at damage areas leads to an excessive amount of oxidative stress, which is considered the main reason for secondary neurodegeneration [24]. Besides, oxidative stress was believed to play the main role in establishment of neurodegenerative diseases by inducing excessive ROS [25].

Oxidative stress arises from an imbalance between the generation rate of ROS/RNS and the ability of the antioxidative defense system to detoxify these reactive species, causing the average level of oxidants to significantly exceed the resting level [15, 26]. The pathological injury caused by oxidative stress includes DNA damage, lipid peroxidation, protein dysfunction, and activation of harmful signaling pathways [11]. To control oxidative stress damage and restore the original state of redox homeostasis, the importance of antioxidant systems cannot be emphasized enough.

#### 2.1.2 Antioxidative Systems

Antioxidants can counteract free radicals and neutralize oxidants. The general endogenous antioxidative systems consist of enzymatic antioxidants and non-enzymatic anti-oxidants.

The enzymatic anti-oxidants contain superoxide dismutase (SOD), catalase (CAT), glutathione peroxidase (GPx), and thioredoxin (Trx) [27]. SOD can convert $O_{2}^{\cdot -}$to oxygen and H_2_O_2_. Three isoforms of SODs have been found in mammals: cytosolic CuZn-SOD (SOD1), mitochondrial Mn-SOD (SOD2), and extracellular SOD (SOD3) [28]. CAT represents the enzyme involved in the reductive depletion of H_2_O_2_ to molecular oxygen and water [29]. GPx, a selenium-containing enzyme, can use glutathione (GSH) as a reductant to catalyze H_2_O_2_ or organic hydroperoxides into water or corresponding alcohols, respectively[27, 30]. The thioredoxin system, which includes thioredoxin (Trx), thioredoxin reductase (TrxR), and NADPH, is intimately associated with the glutathione system, which contains glutathione (GSH), glutathione reductase (GR), and NADPH. These two cellular disulfide reductase systems are critical antioxidative systems in the brain and regulate various redox signaling in the central nervous system [31].

The non-enzymatic antioxidants comprise Vitamin C, Vitamin E, carotenoids, glutathione (GSH), reduced coenzyme Q, flavonoids, and other antioxidants. The details of these non-enzymatic anti-oxidants can be found in a wealth of literature [10, 27, 29]. Later in this review, we will present melatonin, an endogenous non-enzymatic antioxidant.

**Figure 1.**
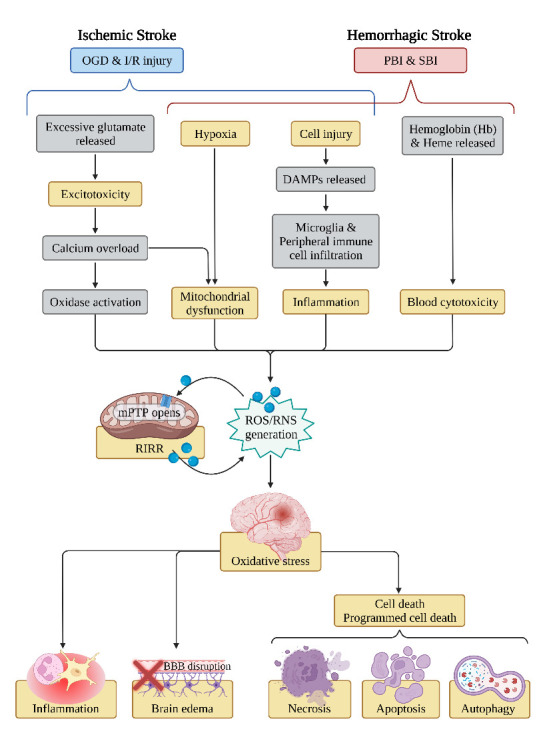
The sources and damage of oxidative stress in stroke. *Abbreviations:* OGD: oxygen and glucose deprivation, I/R: ischemia-reperfusion, PBI: primary brain injury, SBI: secondary brain injury, DAMPs: damage-associated molecular patterns, ROS: reactive oxygen species, NOS: reactive nitrogen species, mPTP: mitochondrial permeability transition pore, RIRR: ROS-induced ROS release, BBB: blood-brain barrier.

### 2.2 Oxidative Stress in the Pathophysiology of Stroke

The brain is highly vulnerable to oxidative stress due to its high oxygen consumption (accounting for more than 20% of the whole body at the resting state) and its rich content of iron and unsaturated lipids [3, 32]. During stroke, a combination of multiple factors is responsible for the over-production of ROS/RNS, which cause oxidative damage to the brain’s structure and function. The following parts briefly review the pathophysiological mechanisms of free radical production and injury after stroke (Fig. 1).

#### 2.2.1 Sources of Free Radicals in Stroke

In ischemic stroke, three distinct phases of ROS generation have been identified in hippocampal and cortical neurons according to a previous study. These three stages are oxygen and glucose deprivation (OGD), activation of xanthine oxidase (XO), and reperfusion [33]. In the first stage, the immediate blocking of cerebral blood flow caused by ischemic stroke deprives the oxygen and glucose supply to the brain tissue. Then, the dysfunction of energy-dependent ion pumps and channels leads to the massive release and impaired reuptake of excitatory glutamate, allowing excess glutamate to accumulate, ultimately leading to excitotoxicity [3, 34]. Multiple downstream signaling pathways could be overactivated by excitotoxicity. Notably, the excessive glutamate can induce calcium overload via N-methyl-D-aspartate receptors (NMDARs) [35]. The increased calcium concentration within cells or organelles leads to activation of a series of pathophysiological cascades (e.g., mitochondrial dysfunction, ER stress, activation of cyclooxygenases and phospholipases, and activation of NO synthase). These signaling cascades result in an enhanced generation of free radicals [11, 36, 37]. In the second stage, the catabolism of intracellular ATP following OGD leads to the conversion of adenine nucleotides to hypoxanthine and xanthine, and these products are substrates for XO to generate ROS [33, 38]. The final ROS/RNS-producing stage is associated with reperfusion. Substantial evidence has shown that the restoration of blood flow reperfusion, which carries a flood of molecular oxygen, calcium ions, and leukocytes, causes a series of damage via excessive ROS/RNS production and inflammatory response [15, 39, 40]. In addition, other ROS-generated mechanisms, such as the activation of NADPH oxidase in immune cells during inflammation (called neutrophil respiratory burst)[41, 42], and already elevated ROS in mitochondria induce a sustained opening of mitochondrial permeability transition pores (mPTP), leading to a new ROS burst, known as ROS-induced ROS release (RIRR) [43, 44].

The injury mechanisms of hemorrhagic stroke are categorized as either primary or secondary brain injury (SBI). The former concerns the mass effect primarily caused by hematoma expansion, whereas SBI involves more pathophysiological events, including the generation of free radicals. After the onset of hemorrhagic stroke, the extravasated blood components (primarily erythrocytes and plasma proteins) from the ruptured blood vessels and the damage-associated molecular patterns (DAMPs, including nucleic acids, proteins, lipid mediators, ATP, etc.) from the injured tissues initiate SBI [45]. DAMPs not only produce a directly cytotoxic insult on adjacent brain cells, but also activate microglia, the brain-resident phagocytes, by binding to specific pattern recognition receptors (PRRs) on the cell surface [46, 47]. The activated microglia produce and release pro-inflammatory cytokines and chemotactic factors, such as TNF-α, IL-1β, and IL-6, which assist in recruiting peripheral immune cells into the CNS [45, 48, 49]. The activated microglia also contribute to a large release of free radicals [50, 51]. The infiltration of neutrophils and mononuclear phagocytes triggers sterile inflammation in the damaged area. Although these inflammatory cells embody a repairing function by scavenging inflammatory mediators and cellular debris, the overpowering immune response worsens neurological prognosis through various damaging mechanisms, including oxidative stress [52]. In addition, DAMPs activate the complement system. The activated complement cascade triggers the formation of the membrane attack complex (MAC), which forms a pore in the cell membrane and leads to erythrolysis [46, 53]. Erythrolysis causes the release of hemoglobin (HB), which subsequently degrades into heme and iron. HB and its degradation products initiate the generation of free radicals via Fenton/Haber-Weiss reactions, leading to oxidative damage of brain cells [45, 54]. One study found that erythrolysis occurs within 24 h of ICH [55], suggesting the importance of early prevention of hemolysis-inducing oxidative damage. In ICH rat models, researchers have demonstrated the beneficial role of some agents, such as N-acetylheparin (a complement inhibitor), aurintricarboxylic acid (a membrane attack complex inhibitor) [56], and trehalose [57], in reducing hemolysis and mitigating subsequent brain damage. Moreover, many ROS/RNS-generating pathways co-exist in both ischemic and hemorrhage strokes.

#### 2.2.2 Damage of Oxidative Stress after Stroke

The role of oxidative stress in stroke can be summarized into three main components: inflammation, brain edema, and neuronal death.

As mentioned above, inflammatory cells, including microglia and infiltrating neutrophils/macrophages, are sources of ROS/RNS. Notably, ROS/RNS can activate these inflammatory cells and exacerbate the inflammatory response, in turn giving rise to a vicious cycle. Firstly, oxidative stress can enhance inflammation by releasing DAMPs through their direct damaging effects on brain tissue [58]. In addition, excess ROS/RNS act as critical signaling molecules in the activation of nuclear factor kappa-B (NF-κB), a transcription factor that induces the expression of multiple pro-inflammatory factors that activate and recruit immune cells [45, 58, 59]. Subsequently, accumulating immune cells release free radicals and proteolytic enzymes in lesions, resulting in inflammation damage.

Multiple pathological processes are responsible for post-stroke brain edema, including oxidative stress. ROS/RNS plays a role in both cytotoxic and vasogenic edema. Cytotoxic edema is associated with the function of ion transporters, and these membrane complexes, such as Na/K-ATPase, Ca^2+^-ATPase, and Na^+^/Ca^2+^ exchanger, could be inhibited by ROS through the peroxidation of membrane phospholipids and modification of proteins [4]. Vasogenic edema partly stems from the destruction of the blood-brain barrier (BBB) caused by the activation of matrix metalloproteinase-9 (MMP-9). The activated MMP-9 degrades the extracellular matrix of the vascular wall and tight junction proteins, leading to BBB disruption [11, 60]. $O_{2}^{\cdot -}$and NO, alongside their potent toxic metabolite, peroxynitrite (ONOO^-^), reportedly activate MMP-9 through directly activating pro-MMP or via the NF-κB pathway [5, 61].

Apart from directly causing neuronal necrosis, ROS/RNS could mediate other programmed cell death processes, such as apoptosis, autophagy, and ferroptosis, which have been widely studied. However, their mechanisms remain incompletely understood.

Apoptosis is a form of programmed cell death that plays a pivotal role in homeostasis [62, 63]. However, the over-activated apoptosis pathways caused by oxidative stress can negatively impact stroke prognosis. Regarding the intrinsic pathways, excess ROS generated within mitochondria lead to the opening of mPTP, resulting in the release of cytochrome c (Cytc) or apoptosis inducing factor (AIF), which then sequentially activates caspases [64, 65]. From the extrinsic pathways speaking, ROS bind within the TNF family of ligands (such as FAS-L and TRAIL) to trigger the activation of downstream caspases [4]. Both intrinsic and extrinsic pathways lead to the activation of caspase proteins, which induce a series of apoptosis-related DNA damage via multiple mechanisms [66]. Besides, ROS also stimulates neuronal apoptosis by activating P53 [67], mitogen-activated protein kinase (MAPK), and apoptosis signal-regulating kinase 1 (ASK1) [27].

Apoptosis is a caspase-dependent process. However, autophagy is a caspase-independent process in which autophagosomes, a bilayer membrane structure sequestering cellular proteins and organelles, can be observed [26]. Similar to apoptosis, autophagy also contributes to homeostasis. According to previous studies, both mitochondrial and ER fragments damaged by ROS are sequestered in autophagosomes to prevent calcium leakage from these organelles, diminishing the death signal strength and suppressing cell death [68]. Meanwhile, abnormal activation of the autophagic pathway may lead to secondary brain damage. During stroke, the overproduction of ROS/RNS is involved in the activation of autophagy [69, 70]. ROS/RNS can trigger mitochondrial outer membrane permeabilization (MOMP) and stimulate the proteolytic activity of autophagy protein-4 (ATG4), thereby activating autophagy [54]. Autophagy not only destroys a large proportion of organelles and leads to a collapse of cellular functions, but also serves as a stimulus to trigger apoptosis and necrosis [62].

Ferroptosis, a recently uncovered regulated form of cell death, is characterized by lipid hydroperoxide accumulation, iron ion dependence, and the absence of apoptotic hallmarks [72]. Although the mechanism of ferroptosis remains unclear, some researchers have reported that ferroptosis is associated with the inhibition of system $X_{C}^{-}$(the glutamate/cystine antiporter) [73] and glutathione peroxidase 4 (GPX4). GPX4 is the only enzyme that can reduce lipid hydroperoxides within biological membranes [74]. When suffering from oxidative stress, the massive production of ROS leads to glutathione depletion. It then causes the dysfunction of glutathione-dependent GPX4 [75], resulting in lipid hydroperoxides accumulating and enhancing ferroptosis. Besides, the iron ion catalyzes the generation of ·OH through the Fenton reaction, and ·OH is quite proficient in causing massive lipid peroxidation, which could further promote ferroptosis or other forms of oxidative damage.

## 3. Melatonin

### 3.1 Melatonin as Antioxidants

Melatonin (N-acetyl-5-methoxytryptamine) is an evolutionarily conserved hormone predominantly synthesized from the pineal gland. This hormone has also been found in other parts of the body, including the retina, skin, gastrointestinal tract, and bone marrow [76, 77]. As a result of its highly lipophilic nature, melatonin can easily pass through all cell membranes, including the blood-brain barrier (BBB) [78]. This ability to cross the BBB paired with its inherent low toxicity as a natural hormone has made melatonin a hot research topic over the past several decades. The pineal gland’s production and secretion of melatonin are controlled by the light-dark cycle, with a maximum level found in the nocturnal phase and a low level in the diurnal baseline phase [79]. During the day, the suprachiasmatic nucleus (SCN, the circadian biological clock) receives neural messages from the retinal cells through the optic nerves, which suppresses the generation of melatonin. When the inhibitory signal from the retinal cells is lifted at night, the SCN promotes melatonin production, and then the pineal gland releases melatonin into the cerebrospinal fluid and blood to regulate the circadian rhythm of the body, such as the sleep-wake cycle [80, 81]. Notably, researchers found that endogenous melatonin levels and the total antioxidant capacity (TAC) in human serum exhibited similar 24-hour variations, both reaching a nocturnal peak at 1:00 am. When the volunteers were given a bright light at night, the melatonin concentration and the value of TAC decreased, demonstrating the antioxidative potential of melatonin [82].

**Figure 2.**
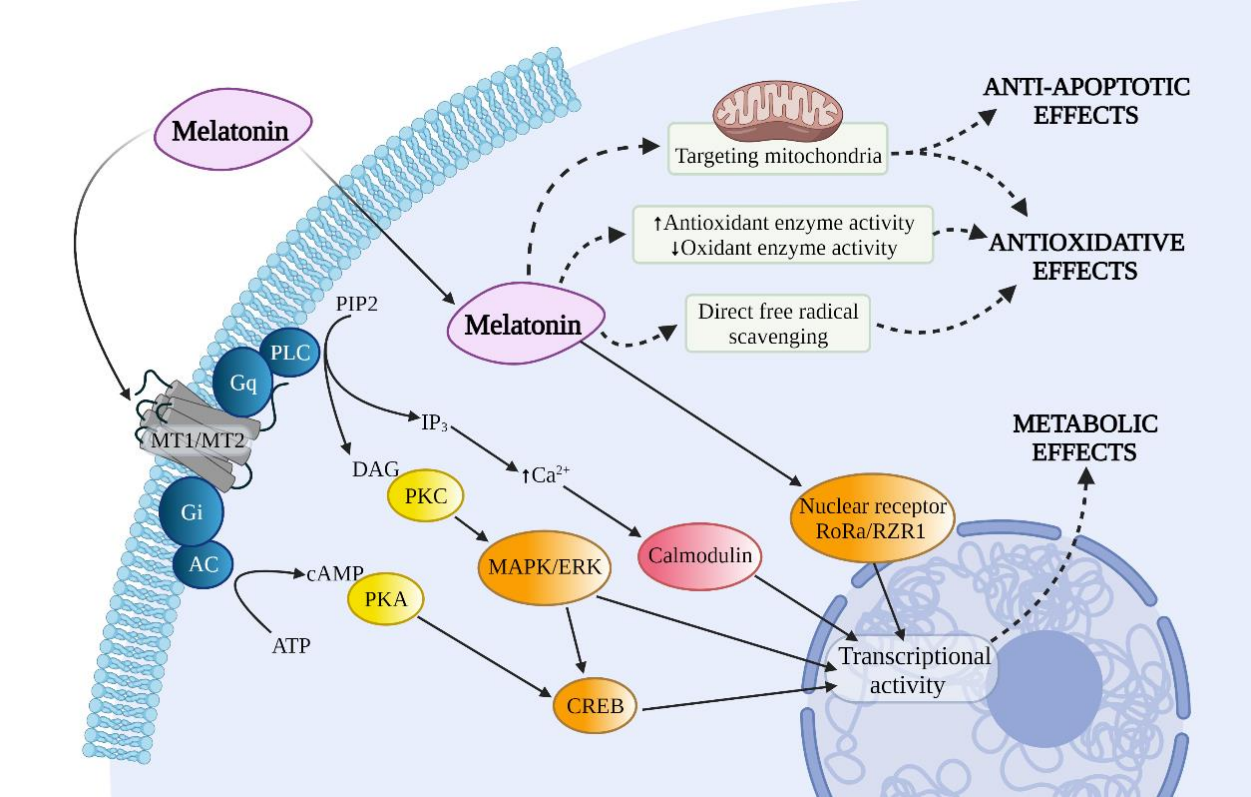
Several pathways and molecular actions of melatonin. *Abbreviations:* MAPK: mitogen-activated protein kinases, ERK: extracellular signal-regulated kinase, CREB: cAMP-response element binding protein, RoRa: retinoid-related orphan receptor alpha, RZR: retinoid Z receptor.

Nearly thirty years have been invested in exploring melatonin’s antioxidative properties[83]. Melatonin can reduce membrane lipid peroxidation, and protein or DNA damage under the condition of oxidative stress [84, 85]. The mechanisms of the antioxidative property of melatonin include: (1) directly scavenging ROS/RNS, (2) activating antioxidative systems, (3) targeting mitochondria (Fig. 2). Firstly, a wealth of research has demonstrated that melatonin is capable of scavenging various types of free radicals, including $O_{2}^{\cdot -}$, H_2_O_2_, ·OH, NO·, and ${\mathrm{ONOO}}^{-}$[81, 86]. Interestingly, one of melatonin’s metabolites, cyclic 3-hydroxymelatonin (c3OHM) [87], alongside its downstream metabolites, N-acetyl-N-formyl-5-methoxykynuramine (AFMK) and N-acetyl-5-methoxykynuramine (AMK), have each been proven to be capable of radical detoxification [88]. Therefore, the ROS/RNS scavenging ability shared by melatonin and its metabolites allows melatonin to target a broader range of oxidizing molecules than classic anti-oxidants [80]. Some researchers believe that melatonin can be considered a prodrug that produces active metabolites (AFMK and AMK) to scavenge both reactive oxygen and nitrogen species [89]. Secondly, melatonin exerts its indirect ROS/RNS scavenging ability to suppress pro-oxidative enzymes [90] and to activate antioxidative enzymes [79]. Moreover, melatonin contributes to maintaining physiological glutathione (GSH) levels, which has a beneficial effect on sustaining redox homeostasis [79]. Thirdly, mitochondria are important targets for melatonin, and some scholars categorize melatonin as a mitochondria-targeted anti-oxidant [80]. In addition to directly scavenging ROS produced in mitochondria, melatonin increases the expression of complexes I and IV of the ETC and limits electron leakage from the ETC, which assist in decreasing the reduction of oxygen molecules to superoxide anion radical ( $O_{2}^{\cdot -}$) [84, 91]. These effects of melatonin help reduce mitochondrial oxidative stress and maintain mitochondrial function and homeostasis, thereby curtailing subsequent apoptotic events and cell death.

Other beneficial effects of melatonin have also been noted. In 1998, melatonin was reported to bind multiple heavy metals [92]. Melatonin’s ability to chelate metals, such as iron, not only blocks the Fenton reaction and inhibits ·OH production, but also assists metallothionein with detoxing transition metals [80]. The anti-inflammatory property is one of the significant functions of melatonin, which indirectly reduces oxidative stress, as the inflammatory response is typically accompanied by free radical generation. Melatonin exerts its anti-inflammatory property through the inhibition of NF-κB activation and through the upregulation of nuclear factor erythroid 2-related factor 2 (Nrf2), among others [93, 94]. In addition, melatonin participates in the anti-apoptotic process. The release of cytochrome c (Cytc), an apoptotic protein, from Ca^2+^-mediated mitochondria can be inhibited by melatonin [95]. Melatonin’s anti-apoptotic capability is also associated with silent information regulator 1 (SIRT1). The upregulation of SIRT1 could increase the anti-apoptotic factor, Bcl2, and decrease the pro-apoptotic factor, Bax, allowing for an increase in the Bcl-2/Bax ratio in parallel with the anti-apoptotic effect [96]. Notably, melatonin may reduce apoptosis and its subsequent damage by regulating mitophagy [97].

### 3.2 Melatonin and Its Receptors

The receptor-mediated mechanism is a significant part of melatonin exerting its potency. In hippocampal slice cultures deprived of oxygen and glucose, ROS production was significantly reduced almost to basal levels using melatonin, and this protective effect was blocked by luzindole, a melatonin receptor antagonist [98], revealing that melatonin exerts part of its neuroprotective functions through melatonin receptors.

Melatonin receptors are widespread in the CNS and peripheral organs [99]. Based on previous literature, the primary physiological actions of melatonin are mediated by stimulation of two high-affinity melatonin receptors, MT1 and MT2 receptors, which belong to the seven-transmembrane G protein-coupled receptor (GPCR) superfamily [7, 100]. Aside from MT1 and MT2 receptors, melatonin has been shown to interact with other intracellular proteins, including nuclear receptor ROR/RZR, quinone reductase 2 (or MT3), and calmodulin [84]. In humans, the MT1 receptor (Kd = 20-40 pM) has a higher affinity for ^125^I-melatonin than the human MT2 receptor (Kd =160 pM) [101]. Notably, these two receptors between species demonstrate considerable differences in affinity in the face of selective MT1/MT2 ligand [100, 102], suggesting that the differences in species must be considered when using animal models for pharmacological experiments. As GPCR, MT1/MT2 receptors could couple to different G proteins (G_i_, G_q/11_, etc.) that mediate the inhibition of adenylyl cyclase (AC), the activation of phospholipase C (PLC), or the inhibition of soluble guanylyl cyclase (GC), leading to a change in numerous secondary messengers, such as cAMP, cGMP, diacylglycerol, arachidonic acid, IP3, and inorganic calcium [103, 104]. In addition, the MT1 receptor stimulates the MEK1/2-ERK1/2 pathway and regulates ion fluxes and specific ion channels [105]. These signaling pathways affected by MT1/MT2 receptors regulate downstream gene expression, thus participating in a range of physiological processes, including cell proliferation, immune responses, and reproductive or metabolic functions [101].

Melatonin’s receptor-mediated neuroprotective effects have been proven in extensive animal studies. In the mouse model of ischemic stroke, both MT1 and MT2 receptors demonstrate their neuroprotective effects by attenuating ischemic insult, and this therapeutic effect of melatonin can be eliminated by melatonin receptor antagonist, luzindole [106, 107]. Furthermore, in newborn mice pups that suffered from hypoxic-ischemic brain injury (unilateral carotid ligation), melatonin can mediate neuroprotective effects via the MT1 receptors, and melatonin can increase the expression of MT1 receptors in the brains of these mice [106]. Besides, MT1 receptors are expressed in neural stem cells (NSC), and MT1 receptors can mediate increased expression of glial cell-line derived neurotrophic factor (GDNF) in NSCs, thereby promoting neuroprotective effects of NSCs [108]. Similarly, MT1 receptors can regulate neural differentiation in amniotic epithelial cells (AEC) in ischemic and oxidative stress injury models. The neuroprotection from AECs would be abolished if MT1 was blocked [109]. Using the live-animal imaging system, researchers have found that melatonin can reduce the production of free radicals via MT2 receptors in mouse models of ischemic stroke [107]. The activation of MT2 receptors also promotes neurogenesis, cell proliferation, and the integrity of BBB [107]. Moreover, it also attenuates the activation of microglia and astrocytes against ischemic injury [110, 111].

## 4. The Neuroprotective Actions of Melatonin in Stroke Model

In experimental models of stroke, consisting of ischemic stroke, hemorrhagic transformation, ICH, and SAH, melatonin has demonstrated its neuroprotective effects by improving the mortality and neurological scores of animals. The following sections will survey the potential mechanisms under the melatonin-mediated neuroprotection found in recent studies (Table 1).

**Table 1:** The main findings of melatonin-mediated neuroprotective mechanism in stroke models.

Model	Drug administration	Main findings	References
120-min transient MCAO, SD rats	10 mg/kg, i.p., consecutive 7 days before ischemia	Pretreatment of melatonin can inhibit ER stress-dependent autophagy	Feng et al. (2017)[115]
MCAO, SD rats	5 mg/kg, i.p., at 30 min before ischemia	Attenuate oxidative stress-associated MAPK (p-P38/p-JNK), and enhance antioxidative systems	Ling et al. (2020)[117]
30 or 90 min transient MCAO, C57BL/6 mice	4 mg/kg, i.p., at the onset of reperfusion	Decrease infarct volume and apoptosis via PI3K/Akt pathway; inhibiting p53 phosphorylation through PI3K/Akt pathway	Kilic et al. (2017)[118]
5-day transient MCAO, C57BL/6 mice	20 mg/kg, i.p., after ischemia and before reperfusion	Attenuate the I/R injury and apoptosis by regulating SIRT3 pathway	Liu et al. (2019)[119]
Distal MCAO (dMCAO), C57BL/6 mice	20 mg/kg, i.p., at 0 and 24 h after ischemia	Regulate microglia/macrophage polarization toward anti-inflammatory phenotype through STAT3 pathway	Liu et al. (2019)[120]
5-min transient global cerebral ischemia (tGCI), Mongolian gerbils	10 mg/kg, i.p., once daily at 6:00 pm for 25 days from 5 days after ischemia/reperfusion.	Improve cognitive deficit by upregulating ERK1/2 expression in oligodendrocytes and restoring glutamatergic synapses	Chen et al. (2018)[121]
90 min transient MCAO with 24 h reperfusion, C57BL/6 mice	15 mg/kg, i.p., at the onset of reperfusion	Alleviate I/R-induced BBB disruption by modulating α7nACh receptor	Chen et al. (2021)[134]
Hyperglycemic MCAO Model, SD rats	150 mg/kg, i.p., at 2 h after MCAO	Attenuate hyperglycemia-enhanced HT by inhibiting ROS/NLRP3 pathway	Shao et al. (2021)[135]
ICH (autologous blood injection), SD rats	5 mg/kg, i.p., 1, 24, and 48 h after ICH	Exert neuroprotective function by inhibiting BBB disruption, inflammation, oxidative stress, mPTP opening, and increasing the ratio of Bcl-2/Bax	Wang et al. (2018)[137]
ICH (collagenase injection), Wistar rats	15 mg/kg, oral, 1 h before or 6 h after the lesion for 7 days	Enhance electrical responsiveness in cerebral cortex by protecting corticospinal tract from oxidative stress	Ueda et al. (2014)[138]
ICH (autologous blood injection), C57BL/6 mice	20 mg/kg, i.p., 30 min before ICH	Inhibit microglial necroptosis via the A20/RIP3 pathway	Lu et al. (2019)[139]
ICH (autologous blood injection), SD rats	150 mg/kg, i.p., 1 hour after ICH	Suppress the expression of ATF6 and CHOP in both protein and mRNA levels	Xu et al. (2018)[140]
Hyperglycemic ICH Model (autologous blood injection), SD rats	5, 10, or 15 mg/kg, i.p., 2, 24, and 48 h after ICH	Attenuate the hyperglycemia-induced brain injury via the PPARδ/PGC-1α pathway	Liang et al. (2020)[144]
Endovascular perforation SAH model, SD rats	150 mg/kg, i.p., 2 h after SAH	Reduce neuronal cell death and inhibit NLRP3 inflammasome activation by promoting mitophagy	Cao et al. (2017)[150]
Endovascular perforation SAH model, C57BL/6 mice	150 mg/kg, i.p., 2 and 12 h after SAH	Attenuate EBI via the melatonin receptor/SIRT1/NF-κB signaling pathway; Inhibit apoptosis	Zhao et al. (2017)[151]
Endovascular perforation SAH model, C57BL/6 mice	150 mg/kg, i.p., 2 and 12 h after SAH	Attenuate EBI by upregulating the expression of SIRT3; Inhibit apoptosis	Yang et al. (2018)[152]
Endovascular perforation SAH model, C57BL/6 mice	150 mg/kg, i.p., 2 and 12 h after SAH	Attenuate EBI by upregulating Nrf2; Inhibit apoptosis; Enhance autophagy	Sun et al. (2018)[153]
Endovascular perforation SAH model, SD rats	5 mg/kg or 10 mg/kg, i.p., 2 h after SAH	Inhibiting both apoptosis and autophagy against EBI through ROS-MST1 pathway	Shi et al. (2018)[154]
Endovascular perforation SAH model, C57BL/6 mice	150 mg/kg, i.p., 2 and 12 h after SAH	Reduce apoptosis against EBI through H19-miR-675-p53 and H19-let-7a-NGF pathway	Yang et al. (2018)[158]
Endovascular perforation SAH model, SD rats	Unknown	Ameliorate post-SAH cerebral vasospasm through H19/miR-138/eNOS/NO and H19/miR-675/HIF1α pathway	Hou et al. (2020)[159]
Endovascular perforation SAH model, C57BL/6 mice	50 mg/kg, i.p., 15 min after SAH	Attenuated EBI via inhibiting NLRP3-induced apoptosis of oligodendrocytes	Liu et al. (2020)[187]

p-P38: phosphorylated-P38, p-JNK: phosphorylated-c-Jun N-terminal kinase, PI3K: phosphatidylinositol 3-kinase, Akt: protein kinase B, I/R: ischemia-reperfusion, SIRT: silent information regulator, STAT3: signal transducer and activator of transcription 3, ERK1/2: extracellular signal-regulated kinase 1/2, BBB: blood-brain barrier, α7nACh: α7 nicotinic acetylcholine, HT: hemorrhagic transformation, ROS: reactive oxygen species, NLRP3: Nod-like receptor protein 3, ICH: intracerebral hemorrhage, mPTP: mitochondrial permeability transition pore, Bcl-2: B cell lymphoma 2, Bax: Bcl-2-associated X protein, RIP3: receptor-interacting protein 3, ATF6: activating transcription factor 6, CHOP: CCAAT/enhancer-binding protein homologous protein, PPARδ: peroxisome proliferator-activated receptor-δ, PGC-1α: peroxisome proliferator-activated receptor-γ co-activator-1α, SAH: subarachnoid hemorrhage, EBI: early brain injury, NF-κB: nuclear factor-κB, Nrf2: nuclear factor erythroid 2-related factor 2, MST1: mammalian sterile 20-like kinase 1, miR: microRNA, NGF: neural growth factor, eNOS: endothelial nitric oxide synthase, NO: nitric oxide, HIF1α: hypoxia-inducible factor-1α

### 4.1 Melatonin and Ischemic Stroke

Ischemic stroke, making up 62.4% of all new strokes in 2019, is the most prevalent type of stroke [1]. A significant proportion of ischemic strokes are caused by clots (thrombotic or embolic) that obstruct blood flow, resulting in ischemic insult in the area of blood supply from the corresponding cerebral artery. In addition to the neuronal necrosis in the central ischemic area, the penumbra, and surrounding the ischemic region, there occurs a series of pathophysiological cascades, such as excitotoxicity, oxidative stress, and inflammation [3, 112]. Rescuing penumbra is imperative for treating ischemic stroke. However, the existence of a therapeutic time window (within 4.5 h of symptom onset) of tissue plasminogen activator (tPA) has limited the application of this thrombolytic drug to most patients[113]. Therefore, as a potential neuroprotective agent for stroke, melatonin has been extensively studied in animal models to determine its protective effects and therapeutic mechanisms [7, 84].

In 2002, Pei et al. reported that the preventive treatment of melatonin reduces the infarct volume in the rat model of middle cerebral artery occlusion (MCAO) [114]. The mechanisms behind the beneficial effect of prophylactic melatonin administration are still being explored to this today. In a study using an experimental rat MCAO model, Feng et al. demonstrated that pre-ischemic melatonin treatment (10 mg/kg, i.p.) could exert neuroprotection by inhibiting ER stress-associated autophagy pathways, including the PERK/ATF4/CHOP pathway and the IRE1/JNK pathway [115]. This neuroprotection includes reducing infarct volume and brain water content, as well as improving neurological score and survival rate during two weeks post-ischemia [115]. This study illustrated the therapeutic possibilities of prophylactic melatonin use in people at high risk of ischemic stroke. However, other studies have shown that activation of ER-stress-induced mitophagy protects against ischemic brain injury [116]. The relationship between melatonin, ER stress, and autophagy in ischemic stroke warrants further investigation. In another experiment of prophylactic melatonin administration, Ling et al. supplied the rats with melatonin (5 mg/kg, i.p.) 30 minutes before they underwent MCAO surgery [117]. Ling et al. confirmed that melatonin could attenuate oxidative stress-associated damage by inhibiting MAPK (p-P38/p-JNK) and by enhancing antioxidative systems (Nrf2/HO-1/Trx) [117].

The effects and mechanisms of the post-stroke melatonin treatment have also been explored. For example, Kilic et al. found that melatonin (4 mg/kg, i.p.) activates the PI3K/Akt pathway to decrease infarct volume and apoptosis in the MCAO model [118]. The PI3K/Akt pathway can inhibit p53 phosphorylation, contributing to neuronal survival [118]. In a study using the 5-day transient MCAO (tMCAO) models, Liu et al. found that intraperitoneal administration of melatonin (20 mg/kg) to mice reduces apoptosis and ischemia/reperfusion injury through the SIRT3 pathway [119]. The application of 3-TYP, a SIRT3 inhibitor, decreased the protective effect of melatonin, causing an increase in infarct volume and the number of apoptotic cells [119]. Moreover, Liu et al. also found that melatonin can upregulate the expression of SIRT3, Foxo3a, Nrf-2, and SOD2 at the transcriptional level [119], and that the relationship of these proteins’ interactions after melatonin treatment warrants further research. Another study reported that melatonin administration (20 mg/kg, i.p.) in the distal-MCAO (dMCAO) models of mice regulates microglia/macrophage polarization toward the anti-inflammatory phenotype through the STAT3 pathway, which alleviates the pro-inflammatory responses and improves functional outcomes [120].

In terms of researching long-term melatonin treatment, Chen et al. used gerbils, a member of the murine family that lacks posterior communicating arteries, in a transient global cerebral ischemia (tGCI) model and administered melatonin (10 mg/kg, i.p.) for 25 consecutive days [121]. Consistent with the previous findings [122], the results of this study demonstrated that long-term melatonin treatment improves cognitive impairment after tGCI. Moreover, this therapeutic effect of melatonin occurred via remyelination by upregulating ERK1/2 expression in oligodendrocytes and by restoring the glutamatergic synapses in the CA1 hippocampal area [121].

### 4.2 Melatonin and Hemorrhagic Transformation

Hemorrhagic transformation (HT) occurs when cerebral blood flow is restored to the damaged vasculature after ischemic stroke, causing bleeding in the area of ischemia. The incidence of HT ranges from 10-40% of patients with ischemic stroke [123, 124], and this phenomenon leads to increased stroke morbidity and mortality [125]. Unfortunately, the delayed application of tPA, the only FDA-approved drug for ischemic stroke treatment, could increase the risk of HT and potentiate neuronal damage [126]. Given the severity of HT and the significance of tPA for ischemic treatment, methods to reduce HT in patients treated with or without tPA are of critical importance.

BBB disruption plays a pivotal role in the pathological processes of HT. Diverse molecular mechanisms, such as oxidative stress, matrix metalloproteinase (MMP) activation, vascular remodeling, and neuroinflammation, as well as several risk factors, including delayed reperfusion, tPA, hypertension, and hyperglycemia, could contribute to BBB breakdown and are potential triggers for HT prevention or treatment [127, 128].

In the early 21^st^ century, Kilic et al. found that the combined treatment of melatonin with tPA not only reduces neuronal damage in mouse models of tMCAO, but it also reverses the tPA-induced brain injury by decreasing iNOS levels and encouraging the PI-3K/Akt pathway [129, 130]. Other subsequent research had successively demonstrated melatonin’s function in HT by using transient MCAO models. Chen et al. reported that melatonin attenuates post-stroke BBB permeability and reduces the risk of HT after tPA therapy in mice [131]. Furthermore, Hung et al. found that melatonin decreases hemoglobin extravasation and MMP-9 expression after ischemia-reperfusion injury in rats, and no significant change in MMP-2 activity was observed [132]. Similarly, Tai et al. found that melatonin-treated mice had reduced activity of MMP-9 and had attenuated hemorrhagic transformation within the infarct when compared to the control group [133]. These previous experiments demonstrated the role of melatonin to antagonize ischemia-reperfusion (I/R) injury in tMCAO models, and supported the fact that melatonin is suitable as an add-on to thrombolytic therapy for ischemic stroke patients.

In recent years, new studies have further explored the role and mechanisms of melatonin for HT prevention or treatment. Chen et al. administrated melatonin (15 mg/kg, i.p.) to 90 min tMCAO mice models and found that melatonin reduces I/R-induced BBB disruption through modulating the α7 subtype of nicotinic acetylcholine receptor (α7nAChR) [134]. This study showed that modulation of α7nAChR by melatonin significantly reduces high mobility group box 1 (HMGB1) secretion from neurons, which subsequently attenuates microglial activation and BBB disruption [134]. Chen and colleagues also reported that melatonin or α7nAChR agonists could increase the expression of CRTC1 and p-CREB to reduce neuronal loss [134]. Shao et al. confirmed that melatonin (preferably 150 mg/kg) alleviates hyperglycemia-enhanced HT in MCAO rat models [135]. They revealed that melatonin could reduce the inflammatory reaction, infarction, and hematoma volume by scavenging ROS and inhibiting NLRP3 inflammasome [135].

### 4.3 Melatonin and Intracerebral Hemorrhage

Intracerebral hemorrhage (ICH) is a severe public health issue with a high mortality rate, constituting 27.9% of all stroke types [1]. Apart from the primary brain injury caused by mass effect from the accumulating hematoma [53], secondary brain injury (SBI) has been perceived as a potential therapeutic target for ICH. The mechanisms behind SBI, which involve inflammation, oxidative stress, and apoptosis/autophagy, activate a series of pathophysiological cascades, resulting in pathological events, such as BBB disruption, brain edema, and neuronal damage [136]. Consequently, if there were methods to inhibit or regulate the above pathophysiological processes, it would have a positive effect on the prognosis of ICH.

As a pleiotropic molecule, melatonin exhibits multiple protective benefits against brain injury in *in vivo* experiments of ICH. Wang et al., using a rat model induced by autologous blood injection, found that the administration of melatonin (5 mg/kg, i.p.) at 1, 24, and 48 hours after ICH attenuates neurological behavior impairment and reduces brain water content [137]. Wang et al. reported that melatonin inhibits oxidative stress, inflammation, and apoptosis (in the hippocampal CA1 region), attenuates damage of mitochondria and BBB, and promotes the expression of antioxidative enzymes [137]. However, this study did not reveal the deeper molecular mechanisms behind these protective functions, nor did it explore which effect is the primary source of melatonin-derived neuroprotection. In a collagenase-induced model of ICH, Ueda et al. discovered that oral administration of melatonin (15mg/kg) enhances the cerebral cortex’s electrical responsiveness and improves rats’ motor dysfunctions [138]. Ueda et al. also considered that melatonin could protect oligodendrocytes and astrocytes around the lesion from oxidative stress, further reducing corticospinal tract damage [138].

A20, a regulatory protein from the NF-κB signaling pathway, has been proven to be one of the mechanisms by which melatonin functions in a mouse model of ICH [139]. In this study, Lu et al. found that a substantial amount of microglia undergo necroptosis within 72h of ICH, and they demonstrated that melatonin (20 mg/kg, i.p.), when administered to mice 30 minutes before ICH induction, inhibits microglial necroptosis through the A20/RIP3 pathway, which contributes to reducing cell death, inflammation, and mitochondrial damage [139]. With a short-term and high-dose administration of melatonin (150 mg/kg, i.p., one h after ICH) in an experiment, Xu et al. found that the ATF6/CHOP pathway and its downstream apoptotic response were suppressed [140]. However, as the activation of ATF6 could be promoted by ER stress [141], the question of whether melatonin inhibits the ATF6/CHOP pathway directly or regulates ER stress to suppress ATF6 via other pathways indirectly deserves further exploration. In addition, hyperglycemic patients are more likely to have poor stroke outcomes [142, 143]. Liang et al. reported that hyperglycemia exacerbated neuronal apoptosis of rats in the experimental hyperglycemic ICH models [144]. They also demonstrated that melatonin (preferably 10 mg/kg, i.p.) could attenuate hyperglycemia-induced brain injury through the PPARδ/PGC-1α pathway [144].

### 4.4 Melatonin and Subarachnoid Hemorrhage

Subarachnoid Hemorrhage (SAH) is defined as bleeding into the subarachnoid space, the area separating the arachnoid membrane and the pia mater of the brain or spinal cord [145]. According to a global statistic, SAH accounts for 9.7% of all new strokes in 2019 [1]. Moreover, SAH is characterized by a high mortality rate (approximately 35%) and a poor prognosis (only 30% of patients could return to independent living) [146]. The pathophysiological events of SAH are complex and can be broadly classified into two categories—early brain injury (EBI) and delayed brain injury (DBI) [147]. EBI refers to the acute pathological events that immediately begin when the blood is bleeding into the subarachnoid space, and lasts 72 h until the development of cerebral vasospasm (CVS) [148]. With the elevation of intracranial pressure (ICP) and the depression of cerebral perfusion pressure (CPP) and cerebral blood flow (CBF) after SAH occurs, a series of other pathological events are triggered (e.g., excitotoxicity, ionic imbalance, oxidative stress, inflammation, cell death, etc.). These factors constitute the EBI and contribute to its deterioration [147, 149]. In addition, a large proportion of patients suffer from DBI even though they have had their aneurysm effectively treated. The possible causes of DBI include CVS, microcirculatory dysfunction, and micro-thrombosis [147]. With advances in understanding the pathophysiology of SAH, the EBI and DBI share some common pathways in their occurrence and progression [149]. The therapy that aims to intervene in the molecular cascades of EBI may attenuate the delayed neurological dysfunction and improve the long-term prognosis of patients [149]. Melatonin, as a pleiotropic molecule, has been widely explored for its mechanism of action in SAH models.

Consistent with the positive effects of melatonin in other stroke models, melatonin also regulates the cell death processes (e.g., apoptosis, autophagy, and mitophagy) in SAH models. Cao et al. found a rise in proteins associated with mitophagy after melatonin treatment (150 mg/kg, i.p.), and the enhanced mitophagy could subsequently attenuate pro-inflammatory cytokine secretion by inhibiting NLRP3 inflammasome activation [150]. As a result, melatonin attenuated brain edema and neurological dysfunction after SAH. However, this beneficial effect of melatonin could be reversed with pretreatment using 3-MA, an autophagy inhibitor [150].

Several studies found that the melatonin treatment of mice protects the brain from EBI following SAH, including the alleviation of brain edema and improvement of neurological deficits, and these studies revealed that melatonin inhibits SAH-induced neuronal apoptosis [151-153]. Zhao et al. reported that melatonin (150 mg/kg, i.p.) increases expression of Sirt1 and Bcl-2, but decreases expression of NF-κB and Bax. Furthermore, they reported that the increased Sirt1 also regulates the expression of Bcl-2 and Bax. The combination of all these elements results in an anti-apoptotic effect of melatonin [151]. However, this neuroprotective effect was abolished by luzindole or Sirt1 siRNA, suggesting that the melatonin receptor/SIRT1/NF-κB signaling pathway is a protective mechanism of melatonin [151]. Similar to the former research, Yang et al. found that melatonin treatment in mice (150 mg/kg, i.p.) inhibits neuronal apoptosis in the brain cortex, significantly upregulates levels of Bcl-2, downregulates levels of Bax and SOD2, and cleaves caspase-3 [152]. Furthermore, Yang et al. confirmed that melatonin enhances expression of Sirt3 protein levels and the *SIRT3* gene, which may be a regulatory site of melatonin [152]. The repression of SAH-induced neuronal apoptosis from melatonin was also observed by Sun et al. [153]. Notably, they found that the melatonin administration (150 mg/kg, i.p.) enhances SAH-induced autophagy against EBI, and that melatonin could upregulate Nrf2 to promote autophagy [153]. Moreover, Shi et al. found that the melatonin treatment (5 or 10 mg/kg, i.p.) attenuates SAH-induced EBI by suppressing both apoptosis and autophagy, and that the underlying mechanism for this phenomenon involves one protein, mammalian sterile 20-like kinase 1 (MST1) [154]. Melatonin clears excess ROS and reduces MST1 cleavage by ROS, allowing MST1 to maintain the bound state of Bcl-2/Beclin 1 complex, thereby maintaining the inhibitory effect of Bcl-2/Beclin 1 complex on autophagy, and this process is referred to as the ROS-MST1 pathway by Shi et al. [154]. Although most studies have confirmed the suppressive effect of melatonin on apoptosis in the SAH model, the impact of melatonin on autophagy remains controversial. Some previous studies have reported the dual regulatory effects (both stimulatory and inhibitory roles) of melatonin on autophagy [155, 156]. The mechanisms behind melatonin-regulated autophagy remain unclear and warrant further research.

H19 belongs to one of the long non-coding RNAs (lncRNAs), which could regulate the expression of gene(s) at transcriptional or post-transcriptional levels and subsequently regulate diverse biological processes [157]. The possible mechanisms of H19 involvement were investigated by several studies using a melatonin-treated SAH rodent model. Yang et al. revealed that treatment with melatonin (150 mg/kg, i.p.) increases the expression of H19 and inhibits apoptosis via H19-miR-675-p53 and H19-let-7a-NGF pathways in the mouse model of SAH [158]. It has been shown that, by upregulating microRNA-675 and downregulating let-7a, H-19 mediates the downstream nerve growth factor (NGF)- and P53-induced apoptosis [158]. In another study, Hou et al. found that attenuation of SAH-induced cerebral vasospasm in melatonin-treated rats is associated with the H19 pathway [159]. Their results suggested that, by regulating H19/miR-138/eNOS/NO and H19/miR-675/HIF1α pathways, melatonin eventually increases expression of eNOS and decreases expression of hypoxia-inducible factor-1α (HIF1α), resulting in amelioration of post-SAH vasospasm [159].

## 5. Clinical Findings and Clinical Trials of Melatonin in Stroke Therapy

Numerous experiments have demonstrated the neuroprotective capabilities of melatonin against stroke. Its effects protect the brain from oxidative stress, inflammation, and neuronal death, alleviate brain edema, and ameliorate post-stroke neurological deficits [84, 160, 161]. Despite the excellent performance of melatonin observed in animal experiments, the clear, definitive clinical evidence proving the therapeutic effect of melatonin in stroke patients remains relatively lacking. However, stroke unfortunately still has limited treatment options. To explore the clinical application of melatonin and to translate it from the laboratory to the clinic, patient-based clinical research and clinical trials are indispensable. This section summarizes recent clinical findings and clinical trials of melatonin in stroke therapy (Table 2).

**Table 2:** The main findings of clinical studies on melatonin and stroke.

Stroke type	Study type/Patient Characteristics	Main findings	References
Ischemic stroke	Unknown; 120 patients (73 patients aged > 60 years)	Concentrations of 6-sulfatoximelatonin have a direct correlation with the severity of stroke. A high level of 6-sulfatoximelatonin indicates post-stroke cognitive impairment in elderly patients.	Kulesh et al. (2016)[162]
Ischemic stroke (malignant middle cerebral artery infarction)	Prospective study; 64 patients (32 non-survivors)	High levels of serum melatonin are associated with the mortality of patients.	Lorente et al. (2018)[163]
Ischemic stroke	Retrospective study; 573 patients (300 patients treated with melatonin, 2 mg/day at 8:00 pm, oral/nasogastric)	Melatonin is a potential prophylactic drug to reduce the prevalence of delirium after stroke.	Mengel et al. (2021)[167]
ICH	Prospective study; 100 patients (46 non-survivors)	High serum melatonin concentrations have an association with the mortality of patients.	Lorente et al. (2019)[164]
Spontaneous ICH	Retrospective study; 117 patients (53 non-survivors)	Serum melatonin levels are higher in non-survivors than in survivors at any moment during the first 7 days of ICH onset and could assist in mortality prediction.	Lorente et al. (2021)[165]
Spontaneous ICH	RCT; 40 patients (20 subjects in the melatonin group, 30 mg/day at night, nasogastric)	Melatonin group has a shorter duration of mechanical ventilation and ICU stay in comparison with the control group.	Dianatkhah et al. (2017)[170]
Traumatic ICH	RCT; 52 patients (26 subjects in the melatonin group, 3 mg/day at 9:00 pm, nasogastric)	Melatonin shortens the duration of mechanical ventilation, reduces morphine consumption, and accelerates the improvement of GCS score.	Soltani et al. (2020)[171]
Aneurysmal SAH	Prospective study; 169 patients	Serum melatonin concentrations are intimately related to the severity of SAH or the outcome of patients.	Zhan et al. (2021)[166]
Aneurysmal SAH	Retrospective study; 181 patients (39 patients received melatonin, 3-6 mg/day at night)	Melatonin group shows no significant differences in the mortality or the incidence of DCI versus the control group.	Lin et al. (2021)[172]

ICH: intracerebral hemorrhage, ICU: intensive care unit, RCT: randomized controlled trial, GCS: Glasgow Coma Scale, SAH: subarachnoid hemorrhage, DCI: delayed cerebral ischemia

The question of whether the prognosis of stroke patients is related to their melatonin levels has been explored by several researchers. Russian researchers measured the concentration of one melatonin metabolite (6-sulfatoximelatonin) in the acute phase of ischemic stroke patients and evaluated their cognitive impairment with neuropsychological tests [162]. The result of this study revealed a direct correlation with the severity of the ischemic stroke and the concentration of 6-sulfatoximelatonin [162]. Similarly, in a study of patients suffering from malignant middle cerebral artery infarction, Lorente et al. found that non-survivors have significantly higher serum levels of melatonin, total antioxidant capacity, and malondialdehyde than survivors, which indicates that the serum melatonin levels are associated with mortality of patients [163]. Consistent with the studies about ischemic stroke, Lorente et al. also investigated the serum melatonin levels of ICH patients with different prognoses, and they found an association between high serum melatonin concentrations and mortality in patients with ICH [164]. In another study, Lorente et al. compared the differences in serum melatonin levels between surviving and non-surviving patients with severe ICH. The serum melatonin levels were found to be higher in non-survivors than in survivors at any moment during the first seven days of ICH onset, meaning that the concentrations of serum melatonin may assist in mortality prediction [165]. However, this study failed to provide a quantitative indication of serum melatonin on predicting mortality, which may require larger-scale statistics and further analysis. In a prospective study concerning patients with aneurysmal SAH, Zhan et al. found a close correlation between serum melatonin concentrations and severity of SAH or outcome of patients. They suggested that patients with higher serum melatonin concentrations are more likely to have a poor prognosis [166]. Overall, these clinical studies demonstrated that serum melatonin might be a potential predictor of mortality and functional prognosis after stroke. Although the mechanism behind the association between melatonin levels and stroke’s severity remains unclear, some researchers speculate that patients with more severe brain damage can subsequently trigger more severe oxidative stress, which is the origin of higher oxidant species production and higher serum melatonin levels (the activation of the endogenous antioxidative system attempting to maintain redox homeostasis)[164]. Therefore, endogenous melatonin may rise as a secondary factor from the primary brain injury.

The clinical applications of melatonin for stroke are still being explored. In a trial exploring melatonin supplementation and post-stroke delirium (PSD), Mengel et al. reported that prophylactic administration of melatonin (2 mg per day at night) in ischemic stroke patients significantly reduces the prevalence of PSD when compared with the control group [167]. Furthermore, the lack of association between melatonin treatment and delayed PSD onset or reduced PSD duration indicates a possible therapeutic potential of melatonin as a preventive rather than ad hoc treatment strategy [167]. Benzodiazepines and opiates are widely used to address sedation and analgesia for patients in the ICU; however, their side-effect of respiratory depression may prolong the duration of mechanical ventilation, and may be detrimental to prognosis [168, 169]. Melatonin, different from these conventional sedative agents, was being explored in its potential application for ICH patients in the ICU. In the study by Dianatkhah et al., patients treated with melatonin (30 mg per day at night) had a shorter duration of mechanical ventilation and ICU stay versus the control group [170]. Another important finding by Soltani et al. was that the supplementation of melatonin (3 mg per day at night) not only shortens the duration of mechanical ventilation, but also reduces morphine consumption [171]. Besides, Soltani et al. reported a faster rise in GCS scores in melatonin-treated patients than in the control group, and GCS scores on the 6^th^ day were significantly better in the melatonin group versus the control group [171]. Melatonin may contribute to the recovery of stroke patients in the ICU, and multi-center prospective studies are warranted to confirm this finding. Although experimental models of SAH have demonstrated beneficial functions of melatonin, Lin et al. failed to observe significant differences between the melatonin-treated and control groups in patients with SAH, either in terms of mortality or the incidence of delayed cerebral ischemia (DCI) [172]. The limitations of this retrospective study are that the dose of melatonin was prescribed to improve sleep (may not be suitable for researching neuroprotection), and other important pathological processes (such as EBI) were not being considered. Therefore, more rigorously designed prospective studies with larger cohorts are still needed to validate the possibility of melatonin application in SAH.

## 6. Conclusion and Perspectives

In this review, we review the hazards of oxidative stress in stroke (Fig. 1). As a low-toxicity antioxidant with neuroprotective effects, melatonin can reduce oxidative stress, inflammation, brain edema, and neuronal death through direct and indirect effects, as well as through mitochondrial and MT1/MT2 receptor pathways (Fig. 2). Additionally, we summarized the updated advances of melatonin in stroke-based research from the most recently published preclinical experiments and clinical studies.

The recent preclinical experiments confirm the concept that melatonin treatment can significantly improve outcomes and neurobehavioral dysfunction in animal models of stroke, which is consistent with the conclusions from the previous review by Wu et al.[8] and two meta-analyses on experimental hemorrhagic stroke [173, 174]. In studies related to ischemic stroke and HT, melatonin exerted similar functions in reducing I/R injury and BBB disruption, such protective effects on the neurovascular unit make melatonin a possible therapeutic agent for acute ischemic stroke in humans. Notably, recent preclinical studies in stroke models have primarily focused on exploring the underlying mechanisms of melatonin (Table 1) rather than merely confirming whether there is a beneficial effect. More specifically, researchers have found several signaling pathways involving proteins, transcription factors, and non-coding RNAs, which may be regulatory targets for melatonin. Besides, melatonin could inhibit neuronal apoptosis, but the mechanism in which melatonin plays a regulatory role in autophagy remains controversial. The aforementioned mechanisms should be further investigated to understand the regulatory targets of neuroprotection and to facilitate the clinical application of melatonin.

Recent clinical studies on melatonin and stroke, although few in number, have made some progress (Table 2). Several studies based on different stroke types have reported an association between high melatonin levels within the body and poor prognosis (i.e., serum melatonin concentrations may be a predictor of mortality after stroke). This conclusion requires large clinical trials to confirm and determine specific ranges of serum melatonin concentrations that predict mortality. Notably, many factors may cause errors in measuring serum melatonin concentrations, which require particular attention from future investigators [175]. Additionally, one retrospective study found that prophylactic administration of melatonin could reduce the prevalence of delirium after stroke. This result may be based on the effect of melatonin on sleep-wake rhythms, which helps expand the application of melatonin in other analogous post-stroke mental and behavioral diseases (e.g., stroke-related dementia). Two small-sample TCR trials reported that melatonin can serve as a sedative adjuvant for post-stroke intubated patients, which led to shorter ventilation times, reduced morphine dosage, and increased CGS scores. Currently, two RCT trials are ongoing to evaluate the efficacy of melatonin in acute ischemic stroke (ClinicalTrials.gov, Identifier: NCT03843008 and NCT01863277). However, current evidence from clinical studies is insufficient to substantiate the protective effects of melatonin in stroke patients.

By comparing the therapeutic time window, administration timing, and dosage of melatonin in basic experiments and clinical studies (Table 1 & Table 2), we discuss possible directions of improvement for future research.

The therapeutic time window of melatonin has some differences between basic and clinical studies. A large proportion of basic experiments have investigated the neuroprotective effects and mechanisms on the acute phase of stroke. In these studies, the therapeutic time window for melatonin tends to be within 1 to 2 hours after insult, and some experiments will re-dose melatonin at 12, 24, or 48 hours after stroke. However, there is still a paucity of clinical studies on melatonin interventions in acute stroke. Therefore, the question of whether melatonin has neuroprotective effects in human cerebrovascular accidents remains unclear. In addition to the direct protective effect, clinical studies are needed to determine whether melatonin can serve as an add-on drug to extend the therapeutic time window for tPA in ischemic stroke, or whether melatonin can reduce the incidence of complications, such as HT, after stroke. We also noted that the long-term treatment of melatonin, either prophylactic or therapeutic, has some positive effects on animal models [115, 121]. This suggests that melatonin may be available as a dietary supplement for high-risk populations to mitigate acute neurological impairment or that it may improve the long-term prognosis of patients with subacute stroke.

In the clinical and preclinical studies presented, patients were often given melatonin treatment at night, yet the majority of animal stroke models did not clarify the timing (day/night) of melatonin administration. However, all mammals, whether humans or rodents, diurnal or nocturnal, have a similar circadian rhythm—pineal melatonin is synthesized and released into the bloodstream at nighttime [176]. In the current clinical studies, since melatonin is often administered long-term to assist in reducing post-stroke complications, such administration requires minimizing the side effects of melatonin in patients. Diurnal administration of melatonin (when it is not present endogenously) results in the induction of fatigue and sleepiness in humans [177]. Therefore, nocturnal administration of exogenous melatonin is consistent with physiological secretion, regulates the normal sleep-wake rhythms, and reduces side effects. Additionally, most preclinical studies focus on the neuroprotective effect of melatonin. Therefore, the timing of dosing is based on the time of stroke onset rather than on circadian rhythms. Moreover, in animal experiments with long-duration melatonin treatment, the melatonin’s circadian effect is also a factor of concern. For example, in a study using melatonin for 25 consecutive days, melatonin was injected into gerbils (a species of diurnal rodent) at 6:00 pm each night [121]. In another study of preventive melatonin use, Feng et al. failed to specify a daily injection time [115].

Notably, melatonin may have different circadian effects on diurnal and nocturnal rodents. In diurnal rodents, such as the fat sand rat, melatonin secretion at night leads to a decrease in body temperature, whereas in most nocturnal rodents, the body temperature peaks at night, despite diurnal and nocturnal rodents having the same pattern of melatonin secretion [178]. Melatonin administration to nocturnal rodents increased their body temperature, alertness, and locomotor activity, which in turn promoted their activity. Conversely, the melatonin administration to some diurnal rodents led to a decrease in physical activity and body temperature [179]. Humans, as a diurnal species, are similar to diurnal rodents regarding circadian temperature rhythms and the resting response to melatonin. However, most preclinical studies on melatonin and stroke use nocturnal rodents (C57BL/6 mice or SD rats) as disease models, which may not be the most ideal animal models for studying intervention effects of melatonin. Moreover, the C57BL/6 mice, an inbred strain of mice, are melatonin deficient and lack the rhythmicity in melatonin secretion [176, 180], and stroke models based on melatonin-deficient animals may produce different effects than humans when receiving melatonin intervention. The CBA and C3H mice are the two important commercially available melatonin proficient strains [181]. Using animal models, in which circadian rhythms and endogenous melatonin levels more closely resemble those of humans, would assist in obtaining more human-relevant research information and facilitate the transition from bench to bedside.

The appropriate doses and route of administration are critical to the efficacy of melatonin. In rodent models of acute stroke, melatonin that produces neuroprotective effects is often administered at higher doses and via intraperitoneal injection. Ramos et al. believed that the doses of melatonin to reach an anti-inflammatory/scavenger effect are higher than those applied for the treatment of sleep disturbances [81]. Andersen et al. reviewed the possible side effects and safety of exogenous melatonin in humans; they considered melatonin doses of 1-10 mg as standard medical sleeping aids and that short-term use of melatonin is safe, even in extreme doses [182]. Moreover, oral melatonin in humans has a low bioavailability, and oral administration requires approximately 40 min to reach maximal plasma concentration [183]. In addition, intravenous melatonin in humans can promptly increase plasma levels, and intravenous administration of a high dose of 100 mg melatonin does not produce adverse effects[183, 184]. Therefore, the effectiveness of high-dose intravenous melatonin is promising and could be more appropriate for patients with acute stroke. Attention should be given to the pharmacokinetics of melatonin when designing clinical studies [185, 186].

Overall, melatonin has promising potential as a pleiotropic antioxidant in stroke therapy, and future research is necessary to further explore its value in human stroke. Thus, there are some points we think will be useful for future research. In basic experiments: 1) The functions and mechanisms of melatonin in stroke warrant further investigation. 2) Using animal models with circadian rhythms, melatonin levels, and other physiological environments that are more similar to those of humans can yield information more relevant to humans. 3) Stroke patients often have aging, hypertension, and other risk factors. Using stroke models accompanied with these pathological factors for melatonin experiments provides a more comprehensive assessment of melatonin’s functions. In clinical studies: 1) Studies regarding melatonin treatment in acute stroke are lacking and administering melatonin at high doses intravenously may contribute to neuroprotective function. 2) Melatonin has potential as an add-on agent to thrombolytic therapy, which requires future evidence to confirm. 3) Large-scale and well-designed clinical studies are essential to determine the efficacy of melatonin, and there are possible errors that are concerning (e.g., the problem of measurement errors in serum melatonin, and the interference to endogenous melatonin from nighttime lighting in ICU).
